# Accidental intrathecal injection of tranexamic acid: a case report

**DOI:** 10.1186/s13256-023-03768-6

**Published:** 2023-02-16

**Authors:** Salama A. Harby, Neveen A. Kohaf

**Affiliations:** 1 Anesthesia, Intensive Care and Pain Management Department, Damietta Faculty of Medicine, Al-Azhar University, Damietta, Egypt; 2 Clinical Pharmacy Department, Faculty of Pharmacy (Girls), Al-Azhar University, Tanta, Cairo, 31511 Egypt

**Keywords:** Intrathecal, Seizures, Tranexamic acid, Inhalation anesthesia, Medication error

## Abstract

**Background:**

Tranexamic acid is a well-known antifibrinolytic medication frequently prescribed to individuals with bleeding disorders. Following accidental intrathecal injection of tranexamic acid, major morbidities and fatalities have been documented. The aim of this case report is to present a novel method for management of intrathecal injection of tranexamic acid.

**Case presentation:**

In this case report, a 400 mg intrathecal injection of tranexamic acid resulted in significant back and gluteal pain, myoclonus of the lower limbs, agitation, and widespread convulsions in a 31-year-old Egyptian male with history of left arm and right leg fracture. Immediate intravenous sedation with midazolam (5 mg) and fentanyl (50 μg) was delivered with no response in seizure termination. A 1000 mg phenytoin intravenous infusion and subsequently, induction of general anesthesia was performed by thiopental sodium (250 mg) and atracurium (50 mg) infusion, and the trachea of the patient was intubated. Maintenance of anesthesia was achieved by isoflurane 1.2 minimum alveolar concentration and atracurium 10 mg every 20 minutes, and subsequent doses of thiopental sodium (100 mg) to control seizures. The patient developed focal seizures in the hand and leg, so cerebrospinal fluid lavage was done by inserting two spinal 22-gauge Quincke tip needles, one on level L2–L3 (drainage) and the other on L4–L5. Intrathecal normal saline infusion (150 ml) was done over an hour by passive flow. After cerebrospinal fluid lavage and the patient’s stabilization was obtained, he was transferred to the intensive care unit.

**Conclusions:**

Early and continuous intrathecal lavage with normal saline, with the airway, breathing, and circulation protocol is highly recommended to decrease morbidity and mortality. The selection of the inhalational drug as a sedative and for brain protection in the intensive care unit provided possible benefits in management of this event with medication errors.

## Introduction

Medication errors are a crucial agent leading to morbidity and mortality, especially in developing countries [1]. The most commonly known factors associated with medication errors include labels, location and shape of syringes and ampules, dual-checking paucity, inadequate contact, inattentiveness, and exhaustion of the anesthesiologist [2, 3].

### Tranexamic acid (TXA)

TXA is synthesized from lysine and is known to have an antifibrinolytic action, thus it is used in the management of hemorrhagic conditions. It has a similar action as aminocaproic acid; however, it possesses about tenfold more potency. It was approved in 1986 [4].

### Mode of action

TXA inhibits the activation of plasminogen by binding to many different sites, in a manner that is both competitive and reversible. Four or five sites with low affinity and one with strong affinity, the latter is responsible for its binding to fibrin, are present on this protein. By occupying the essential binding sites, TXA inhibits the disintegration of fibrin, therefore stabilizing the clot and avoiding bleeding [5].

### Pharmacodynamics

TXA is an antifibrinolytic that inhibits the activation of plasminogen to plasmin via a competitive mechanism [6]. TXA binds more strongly to both the strong and weak receptor sites of the plasminogen molecule than aminocaproic acid, in proportion to their relative potency [7]. TXA’s reduction of plasmin synthesis and activity may decrease angioedema attacks in individuals with hereditary angioedema by reducing the activation of the first complement protein (C1) by plasmin [8, 9].

Off-target gamma-aminobutyric acid (GABA) (A) receptor antagonism may contribute to the development of hyperexcitability and convulsions after treatment of TXA [10]. Inappropriate administration or administration during cardiovascular surgery tends to increase the risk, so electroencephalogram (EEG) monitoring for patients with a seizure history should be considered [10].

### Pharmacokinetics

Urinary excretion is the major TXA elimination pathway with > 95% of a given dosage eliminated intact in the urine. The excretion rate depends on the mode of administration; around 90% of an intravenously injected dosage is eliminated within 24 hours [11]. After intravenous injection, the estimated elimination half-life is approximately 2 hours, while the average terminal half-life is 11 hours [11]. Plasma clearance of ranges between 110 and 116 ml/minute [11].

### Toxicity

Serious gastrointestinal manifestations, hypotension, thrombosis, impairment of vision, convulsions, mental state fluctuations, and rash have been reported as symptoms of TXA overdose [12]. TXA is routinely kept near other medications, especially injectable local anesthetics for spinal analgesia. Some local anesthetics have packaging that resembles that of TXA (transparent ampoule with transparent solution), which might lead to major adverse effects if administered in place of the intended intrathecal anesthetic [13].

In this instance, due to the identical look of two separate ampules, TXA was administered instead of hyperbaric bupivacaine for spinal anesthesia, and was accountable for generalized myoclonic seizures and hemodynamic instability.

## Case report

A 31-year-old Egyptian male with American Society of Anesthesiologists (ASA) I physical status, with history of polytrauma since 1 week, had a left arm and right leg fracture. A complete trauma survey was done at the time of admission, with no other injury and acceptable laboratory and radiological investigations.The operation was done first to the fracture on left hand under general anesthesia with no complications. Then after 1 week, the patient was scheduled for an operation on the right leg. While sitting in the L3–L4 interspace, a 22-gauge needle with a Quincke tip was used to deliver spinal anesthesia to the patient. About 120 seconds after receiving an injection of 20 mg (4 ml) of hyperbaric bupivacaine 0.5%, the patient had significant back and gluteal discomfort, followed by myoclonic movements in the lower limbs and a generalized convulsion.

The arterial blood pressure of the patient was elevated to 170/100 mmHg, and his heart rate increased to 120 beats per minute. Immediate intravenous sedation with midazolam (5 mg) and fentanyl (50 μg) was delivered with no response in seizure termination. Phenytoin (1000 mg) was given by intravenous infusion and we subsequently applied the airway, breathing, and circulation (ABC) protocol. General anesthesia was induced by a thiopental sodium (250 mg) and atracurium (50 mg) infusion, the trachea of the patient was intubated, and respiratory parameters on mechanical ventilation were as follows: tidal volume (TV) 500 m/minute, respiratory rate (RR) 16 cycles/minute, fraction of inspired oxygen (FIO_2_) 100%, positive end-expiratory pressure (PEEP) 0 mmHg.

Maintenance of anesthesia was attained by isoflurane 1.2 minimum alveolar concentration (MAC) and atracurium 10 mg every 20 minutes, and subsequent doses of thiopental sodium (100 mg) to control seizures. Although with the above interventions, the patient developed focal seizures in the hand and leg. The attending anesthesiologist had suspicions regarding intrathecal administration of the incorrect medicine after discovering a used TXA ampoule (500 mg/5 ml) in the trash. So, cerebrospinal fluid (CSF) lavage was done by inserting two spinal 22-gauge Quincke tip needles on level L2–L3 (drainage) and the other on L4–L5. Intrathecal normal saline infusion (150 ml) was done in 1 hour by passive flow. During CSF lavage, vital signs were as follows: blood pressure (BP) 140/85 mmHg, pulse 140 beats/minute, and the patient was on mechanical ventilation (MV) with FIO_2_ 100 and oxygen saturation (SO_2_) 98%. Arterial blood gases (ABG) were pH 7.42, PaCO_2_ 37, PaO_2_ 157, and HCO_3_ 24 after CSF lavage. The patient was stabilized, and he was transferred to the intensive care unit (ICU).

About 2.5 hours after the injection, the patient was transferred to the ICU and with volume-controlled ventilation mode, the MV was continued. The patient was put on MV, and thiopental sodium infusion was continued, with 100 mg/hour increased to 300 mg/hour, but the patient developed multiple generalized myoclonic seizures so atracurium was started by loading 50 mg then 5–10 μg/kg/hour as apart of complete general anesthesia. However, the seizures were not controlled so we put the patient on an anesthesia machine and continued MV with isoflurane 1.2 MAC, with thiopental sodium 300 mg/hour and 10 μg/kg/hour atracurium.

A central venous catheter was inserted on the right subclavian vein. The initial postoperative ABG examination showed pH of 7.44, PaO_2_ of 170, PaCO_2_ of 47, and HCO_3_^−^ of 27. The MV parameter was adjusted to produce hyperventilation to target PaCo_2_ of 35–40 mmHg with continuous monitoring with capnogram. A complete blood test indicated no evidence of hepatic, renal, or hematological malfunction. Six hours postoperatively, the patient suffered tonico–clonic seizures of the upper limbs and face, which were managed by a continuous infusion of sodium thiopental (3–5 mg/kg/hour), atracurium 10 μg/kg/hour, isoflurane 1.2 MAC, and 150 mg phenytoin every 8 hours.

Cranial computed tomography revealed no abnormality and fundus examination revealed papilledema, so protective brain strategies continued with mannitol 20% 150 ml every 8 hours for 2 days and isoflurane 1.2 MAC and mild fluid restriction and lasix 20 mg every 12 hours. To avoid ventricular arrhythmia, amiodarone was administered prophylactically at a dose of 10 mg/kg/24 hours for 24 hours. The patient developed sinus tachycardia and his heart rate increased to 150 beats/minute, which was controlled by an amiodarone infusion. After 3 hours from admission to ICU, the patient’s blood pressure decreased, with mean arterial blood pressure below 50 mmHg, and investigations showed normal electrocardiogram (ECG) sinus rhythm. Central venous pressure (CVP) was 10 cm H_2_O, so norepinephrine infusion was started at 0.05 μg/kg/minute to maintain tissue and cerebral perfusion. The mean arterial blood pressure then improved with norepinephrine and maintained above 75 mmHg.

On the second day postoperatively, the sedation began to decrease: first isoflurane to reach 0.6 MAC, then isoflurane was stopped, and thiopental sodium was decreased to 200 mg/hour. The patient developed one-time seizures on the face and upper extremities, so sedation was continued for another 24 hours with decreasing thiopental sodium to 200 mg/hour only. Also, the patient became feverish (39 °C) in the second day so, 1 gm intravenous acetaminophen was given every 6 hours.

Norepinephrine was stopped after 24 hours as mean arterial blood pressure was maintained over 75 mmHg without support.

On the third day after surgery, sedation was discontinued. As the patient’s degree of consciousness improved, he moved his head and upper extremities in response to painful stimuli and absence of deep tendon reflexes in the lower limbs. On the fourth day postoperatively, he opened his eyes in response to voice instructions, followed simple directions, and breathed on his own. The trachea was extubated, and all neurologic examinations were acceptable. The patient was discharged from the ICU on the sixth day and transferred to the ward after 48 hours from weaning from MV. The patient was discharged home with recommendation to follow-up with neurology and to be fully evaluated after 6 months. The patient was monitored at 6-month and 1-year intervals and found to be in excellent condition with no neurological symptoms.

## Discussion

The effects of direct intrathecal injection of TXA are little understood. Intrathecal TXA is a potent neurotoxin that produces neurological sequelae, including refractory seizures and a 50% death rate [13]. In 1980, the severe toxicity of intrathecal TXA was discovered. Since 1988, 21 cases of unintentional intrathecal injection of TXA have been documented, of which 20 were life-threatening and ten were fatal. Sixteen were recorded between 2009 and 2018 [14]. There were also three incidents that happened in South Africa between December 2021 and January 2022, which raises the possibility that the problem is more prevalent than suggested by published case reports [15].

The first incidence of accidental intrathecal injection of 75 mg TXA in an 18-year-old male with ASA I physical condition planned for appendectomy was reported by Wong *et al*. [16]. Four hours following a spinal injection, he had continual motor and sensory loss in his lower extremities, in addition to urinary incontinence. Five and a half hours after the injection, he suffered clonic convulsions that proceeded to a generalized seizure and hyperthermia of 40.5 °C. Following intravenous administration of diazepam and diclofenac, the patient’s convulsions and fever gradually decreased over the following 5 hours, and he recovered without any complications.

In the incident highlighted by Yeh *et al*. [17], a catastrophic result was accompanied with refractory ventricular fibrillation and widespread convulsions following intrathecal injection of 500 mg of TXA. In a further case report, intrathecal injection of 150 mg TXA resulted in a severe outcome in one patient due to refractory ventricular fibrillation [18]. Four days after receiving an intrathecal injection of 400 mg TXA, our patient showed a full recovery with our management.

It is unknown precisely how TXA causes convulsions or ventricular arrhythmia. As demonstrated by the first hypertensive reaction and subsequent ventricular arrhythmia, high dosages of TXA would result in enormous sympathetic discharge described in our case report.

Seizures caused by TXA arise from either direct or regional cerebral ischemia, decreases in systemic cerebral circulation, or inhibition of cortical GABA-A receptors [13]. The incidence of convulsive seizures rose from 1.3% to 3.8% among cardiac surgery patients administered TXA [19].

Upon systemic administration of higher doses of TXA, ischemia, cerebral vasoconstriction, and elevated intracranial pressure also play a role. [20]. It has been demonstrated that isoflurane, a noncombustible halogenated (fluorinate) and lipophilic substance, increases membrane fluidity and activates sphingomyelin hydrolysis [21, 22]. This volatile anesthetic provides short-term neuroprotective effects against focal and global ischemia/hypoxia [21]. Thus, we used isoflurane for brain protection in our case.

Our patient showed significant multiple generalized convulsions, which not responding to continuous intravenous infusion sedation, muscle relaxant, and phenytoin, so we used an anesthetic machine to get the brain protection effect of inhalation anesthesia and decrease the need of a high dose of intravenous sedation as we reached maximum doses. There are some studies that showed some benefit of use of inhalational anesthesia in ICU [23, 24]. In the ICU, inhaled anesthetics are mostly used for sedation, refractory bronchospasm, and status epilepticus that is unresponsive to anticonvulsant medicines [25].

Given the dire prognosis associated with this pharmaceutical mistake, Tsui [26] advocated strongly for considering early lavage. Lavage of the cerebrospinal fluid dilutes and eliminates the medication, potentially by dilution. The removal and replacement of 10 ml of CSF with 10 ml of saline may be repeated up to four times. Alternately, once-repeated removal and restoration of 20 ml has been investigated [27]. Insertion of a spinal catheter may facilitate this procedure and careful asepsis will be required during these procedures. Su [28] used 120 ml for lavage. We used 150 ml to better dilute the TXA and minimize adverse events.

Atracurium has a distinctively different metabolic pathway compared with other commercially accessible nondepolarizing muscle relaxants, employing Hoffman elimination and ester hydrolysis without renal or hepatic involvement to minimize drug interaction [29]. Thus, to control the myoclonic jerks that appeared in the upper limbs and face, atracurium was used as part of complete general anesthesia.

The treatment of convulsions and CSF lavage when performed earlier lead to favorable outcomes [28].

## Recommendations and conclusions

All previously referenced case reports were the result of misreading between TXA and 0.5% hyperbaric bupivacaine ampoules, which appeared identical from the outside. We recommend that essential pharmaceuticals, such as those utilized for spinal anesthesia, have a distinct appearance and packaging, so that the probability of error is minimized. Health care professionals must adhere to the practice of double-checking while examining the ampoule label. Informing anesthesiologists and administrators of the risks of intrathecal TXA through workshops and conferences are also important. Research and development departments in pharmaceutical companies are also invited to develop a strategy to overcome this recurring medication error.

**Fig. 1:**
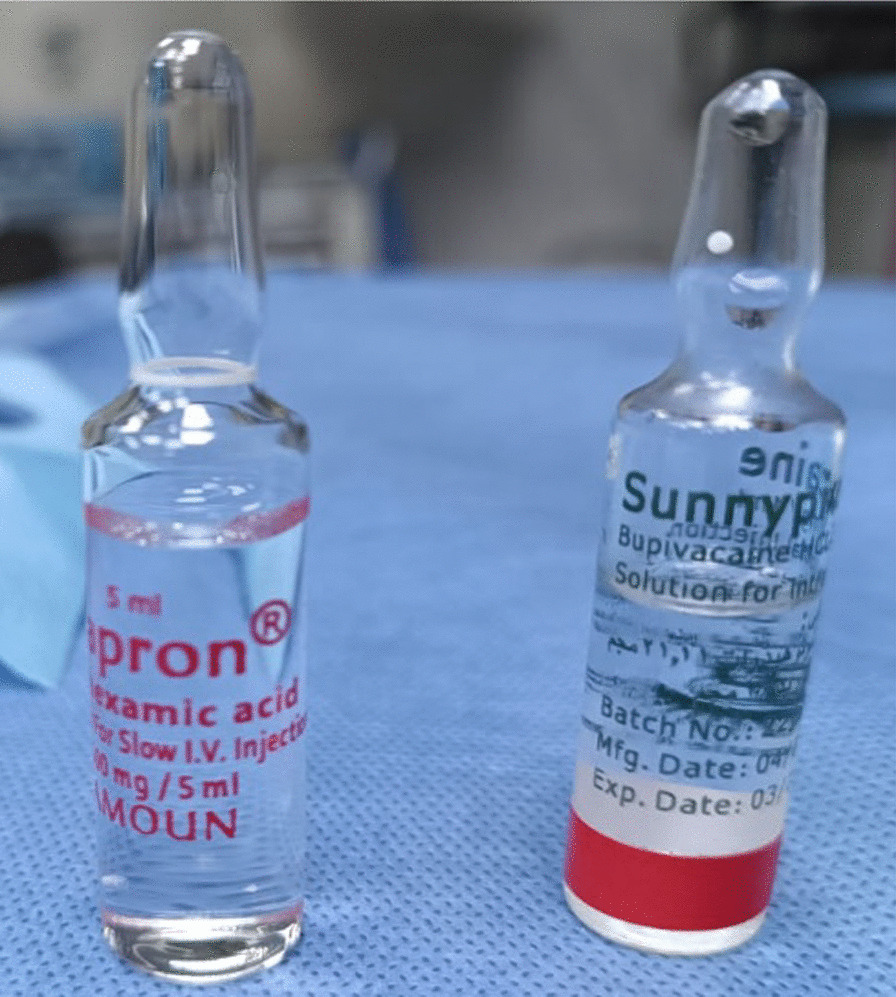
The manufacturer changed the appearance of tranexamic acid and bupivacaine ampoules

It should be mentioned that, due to the frequency of this error, the manufacturer recently altered the look of the two ampoules (Fig. 1).

In conclusion, early and continuous intrathecal lavage with normal saline and the ABC protocol is highly recommended to decrease morbidity and mortality. The selection of the inhalational drug as a sedative and for brain protection in the ICU provided possible benefits in management of this error.

## Data Availability

Not applicable.

## Abbreviations

TXA: Tranexamic acid
IV: Intravenous
CSF: Cerebrospinal fluid
ABC: Airway, breathing, and circulation
ICU: Intensive care unit
C1: Complement 1
GABA: Gamma-aminobutyric acid
ASA: American Society of Anesthesiologists
MAC: Minimum alveolar concentration
ABG: Arterial blood gas
MV: Mechanical ventilation
TV: Tidal volume
RR: Respiratory rate
FIO_2_: Fraction of inspired oxygen
PEEP: Positive end-expiratory pressure
BP: Blood pressure
SO_2_: Oxygen saturation
